# Wisdom, self-empowerment, and distress tolerance in Iranian women with breast cancer: Coping styles and emotion management as mediators: A cross-sectional study

**DOI:** 10.1192/j.eurpsy.2026.11485

**Published:** 2026-07-31

**Authors:** M. Sharifi, C. A. Lewis, M. Firoozi, S. Khani, P. Afkari, A. Seyedmodallalkar

**Affiliations:** 1 Psychology, Leeds Trinity University, Leeds, United Kingdom; 2 Psychology, Islamic Azad University, Bandargaz Branch, Bandargaz; 3 Psychology; 4 Psychology and Educational Sciences, University of Tehran; 5 Psychology, Payam-e-noor University, Tehran; 6 Psychology, Islamic Azad University, Sari Branch, Sari, Iran, Islamic Republic Of

## Abstract

**Introduction:**

There is comparatively only modest research that has examined psychological factors on distress tolerance among women with breast cancer, and associated mediating factors.

**Objectives:**

This study aimed to investigate the effect of wisdom and self-empowerment on distress tolerance among women with breast cancer, considering the mediating roles of coping styles and emotion management

**Methods:**

In this cross-sectional, descriptive-correlational study, 128 women with breast cancer were selected via convenience sampling from the Cancer Research Center at Hospital in Mashhad. Data were collected using validated questionnaires measuring wisdom, self-empowerment, distress tolerance, coping styles, and emotion management. The proposed relationships were analyzed using Pearson’s correlation, multivariate regression, and structural equation modeling (SEM) in SPSS and Smart-PLS.

**Results:**

The findings indicated that wisdom had a significant direct effect on distress tolerance. Furthermore, coping styles served as a significant mediator in the relationship between wisdom and distress tolerance. However, self-empowerment did not exhibit a statistically significant direct effect on distress tolerance, and emotion management did not mediate the relationship between wisdom and distress tolerance.

**Conclusions:**

The results suggest that enhancing wisdom and improving adaptive coping strategies can effectively increase distress tolerance in women with breast cancer. These findings highlight the importance of psychological interventions focused on these constructs in supportive care for cancer patients.

**Disclosure of Interest:**

None Declared

